# Characterising competitive equilibrium in terms of opportunity

**DOI:** 10.1007/s00355-016-1015-7

**Published:** 2017-01-09

**Authors:** Robert Sugden

**Affiliations:** School of Economics and Centre for Behavioural and Experimental Social Science, University of East Anglia, Norwich, NR4 7TJ UK

**Keywords:** Opportunity criterion, Competitive equilibrium, Behavioural welfare economics

## Abstract

This paper analyses alternative profiles of opportunity sets for individuals in an exchange economy, without assuming that individuals’ choices reveal coherent preferences. It introduces the concept of a ‘market-clearing single-price regime’, representing a profile of opportunity sets consistent with competitive equilibrium. It also proposes an opportunity-based normative criterion, the Strong Opportunity Criterion, which is analogous with the core in preference-based analysis. It shows that every market-clearing single-price regime satisfies the Strong Opportunity Criterion and that, in the limit as an economy is replicated, only such regimes have this property.

## Introduction

In normative economics, it has traditionally been assumed that individuals have stable and context-independent preferences over all economically relevant outcomes, and that these preferences are revealed in individuals’ decisions; the satisfaction of these assumed preferences has then been used as a normative criterion. However, research in behavioural economics has uncovered many systematic patterns in individuals’ choices that are not consistent with traditional assumptions about the context-independence of revealed preferences, calling into question the idea that revealed preferences are indicators of welfare. These findings suggest that economics may need a different normative criterion.

In this paper, I develop the approach proposed by Sugden (2004) and McQuillin and Sugden (2012), in which the objects of normative assessment are profiles of individuals’ opportunity sets. McQuillin and Sugden’s papers define an Opportunity Criterion for assessing such profiles. This criterion does not refer to preferences, and can be justified even if revealed preferences are context-dependent or dynamically inconsistent. Those papers show that, in every competitive equilibrium of an exchange economy, the profile of opportunity sets induced by that equilibrium satisfies the Opportunity Criterion.^1^ In the present paper, I ask whether that criterion (or some variant of it) *requires* the profile of opportunity sets to have the properties of competitive equilibrium. I define a stronger version of this criterion, the Strong Opportunity Criterion. I show that every competitive equilibrium satisfies the Strong Opportunity Criterion and that, in the limit as an economy is replicated, the set of opportunity profiles that satisfy the Strong Opportunity Criterion shrinks to the set induced by competitive equilibrium.

Formally, the Strong Opportunity Criterion is closely related to the core, and the convergence theorem that I present is closely related to the Core Convergence Theorem conjectured by Edgeworth (1881/1967) and proved by Debreu and Scarf (1963) and (in a mathematically different form) by Aumann (1964). Conceptually, however, a preference-independent assessment of opportunity profiles is fundamentally different from a conventional preference-based assessment of economic outcomes. My results show that close analogues of canonical welfare theorems can be proved without assuming that individuals act on consistent preferences and without using preference-satisfaction as the normative criterion. This parallelism suggests that the concepts of preference and rational choice are less essential to normative economics than economists have often thought.

## Opportunity in an exchange economy

I define an exchange economy in terms of a nonempty set $I = \{1, {\ldots }, n\}$ of *individuals* with typical element *i* and a set $G = \{1, {\ldots }, m\}$ of infinitely-divisible *commodities* with $m \ge 2$ and typical element *g*. For each individual *i* and commodity $g,e_{i,g }\in [0, \infty )$ represents *i*’s *endowment* of claims on *g*. I assume that, for each commodity $g,\sum _{i\in I }e_{i,g} >0$. The *m*-tuple $\mathbf{e}_{i }= (e_{i,1}, {\ldots }, e_{i,m})$ is *i*’s *endowment vector*. The *n*-tuple $\mathbf{e} = (\mathbf{e}_{1}, {\ldots }, \mathbf{e}_{n})$ is the *endowment profile.* 2 An *exchange economy* is defined by the quadruple $<I, G, \mathbf{e}, \mathbf{f}(.)>$, where **f**(.) is a function, to be defined later, that specifies the choices that individuals make in this economy, given the opportunities that are available to them.

Economic activity takes place in a single period and consists in individuals’ adding or subtracting claims to or from their endowments. For each individual *i*, for each commodity *g*, $q_{i,g} \in [-e_{i,g},\infty )$ denotes *i*’s *acquisition* of *g*, interpreted as the net increase in *i*’s holdings of *g*. An *m*-tuple $\mathbf{q}_{i} = (q_{i,1}, {\ldots }, q_{i,m})$ is an *acquisition vector* for *i*; the universal set of such vectors is denoted by ${\varvec{\mathscr {Q}}}_{i}$. An *n*-tuple $\mathbf{q} = (\mathbf{q}_{1}, \ldots , \mathbf{q}_{n})$ is an *acquisition profile*; the universal set of acquisition profiles (i.e., the Cartesian product ${\varvec{\mathscr {Q}}}_{1} \times {\cdots } \times {\varvec{\mathscr {Q}}}_{n}$) is denoted ${\varvec{\mathscr {Q}}}$. An acquisition profile **q** is *feasible* if, for each good $g, \sum _{i\in I} q_{i,g}= 0$. The set of feasible acquisition profiles is denoted *F* (where $F \subseteq {\varvec{\mathscr {Q}}}$). These feasibility constraints represent the resource limitations of the economy, under the assumption that all goods are initially held by individuals as endowments; they are strict equalities (i.e., there is no free disposal assumption) to allow the model to represent bads as well as goods.^3^

I will say that claims held at the end of the trading period are *consumed* by the individuals who then hold them, but ‘consumption’ need not be interpreted as something that individuals value positively. It represents whatever opportunities and obligations an individual incurs by virtue of holding a claim at the end of the period. Thus, tradable commodities can be goods or bads (or, indeed, goods for some individuals and bads for others). However, commodity 1 (*money*) will be interpreted as a good whose consumption is always valued positively. Because I do not use the concept of preference, this interpretation cannot be stated as an explicit property of the model, but it motivates a concept of ‘dominance’ whose role in my analysis is analogous with that of non-satiation in classical welfare theorems. This analogy will be explained in Sect. 3. Money has a special role in the model as the medium of exchange; the implications of this will be explained in Sect. 4.

My normative analysis applies to any given exchange economy. Throughout the rest of this Section, and throughout Sects. 3, 4 and 5, ‘the’ economy will be taken as fixed, but the results I will prove hold for exchange economies in general. The objects of normative analysis are alternative specifications of the opportunities for acquisition that are available to individuals in the economy.

The opportunities available to an individual *i* are described by a nonempty *opportunity set* $O_{i} \subseteq {\varvec{\mathscr {Q}}}_{i}$. The interpretation is that *i* is free to choose one (and only one) element of this set. Each $\mathbf{q}_{i}\in O_{i}$ is *allowable* in $O_{i}$. A profile $\mathbf{O} = (O_{1}, {\ldots }, O_{n})$ of opportunity sets is a *regime*. An acquisition profile $\mathbf{q}$ is *allowable* in regime **O** if each $\mathbf{q}_{i}$ is allowable in $O_{i}$, with $A(\mathbf{O})$ denoting the set of acquisition profiles allowable in **O**. Notice that an acquisition profile can be allowable even if it is infeasible.

For any individual *i* and any acquisition vector $\mathbf{q}_{i} \in {\varvec{\mathscr {Q}}}_{i}$, $\mathbf{q}_{i}$ is *dominated in* $O_{i}$ if there is some $\mathbf{q}^{\prime }_{i} \in O_{i}$ such that (i) $q^{\prime }_{i,1 }> q_{i,1 }$ and (ii) for each $g \ge 2, q^{\prime }_{i,g }=q_{i,g}$. Given the implicit assumption that consumption of money is always valued positively, a dominated acquisition vector $\mathbf{q}_{i}$ is unambiguously less desirable than the acquisition vector $\mathbf{q}^{\prime }_{i}$ that dominates it. Thus to say that $\mathbf{q}_{i}$ is dominated in $O_{i}$ is to say that, were $\mathbf{q}_{i}$ an element of $O_{i}$, *i* would have no reason to choose it.^4^

I assume that, for each individual *i*, there is a *choice function* $\mathbf{f}_{i}$(.) which assigns a unique chosen acquisition vector $\mathbf{q}_{i}$ to every opportunity set $O_{i} \subseteq {\varvec{\mathscr {Q}}}_{i}$. The acquisition profile that is jointly chosen by individuals from regime **O** is denoted **f**(**O**); **f**(.) is the *joint choice function*. If **f**(**O**) is feasible, **O** is said to be *market-clearing*. Notice that each $\mathbf{f}_{i}$(.) is defined for, and is therefore specific to, the fixed economy. It can be interpreted as taking account of any contextual features of that economy that are potential determinants of *i*’s decisions and that are independent of the opportunities or decisions of other individuals.^5^ Notice that no assumptions are being made about the mechanism that determines what each individual chooses from his opportunity set, only that these choices are predictable, given a full description of the contextual features of the economy.^6^ Thus, choices may be context-dependent.

The model allows patterns of choice that cannot be rationalised by preferences that satisfy conventional conditions. The standard account of rational choice would require that each individual’s choices satisfy the weak axiom of revealed preference. I do not impose this requirement, even for a given economy. Thus, for example, an individual’s revealed preference between two given acquisition vectors may vary according to the opportunity set in which they appear, as in theories of salience (Bordalo et al. 2013) and bad-deal aversion (Isoni 2011; Weaver and Frederick 2012). Because individuals’ endowments are treated as properties of the fixed economy, and because choice functions are specific to that economy, the model imposes no restrictions on how choices respond to changes in endowments. Thus, an individual’s revealed preferences over given bundles of consumption might vary according to his endowments, as in theories of reference-dependent preferences (Tversky and Kahneman 1991; Munro and Sugden 2003).^7^

In interpreting the concept of a regime, it is useful to imagine that exchange is intermediated by some *trading institution*, distinct from the ‘individuals’ of the economy. This institution might be thought of as an ‘auctioneer’ in the sense of Walrasian general equilibrium theory, or as a ‘social planner’ in the sense of modern welfare economics, or as a set of competing profit-seeking ‘traders’ who come to the economy from outside (as in the model of Sugden 2004).^8^ The trading institution offers a set of trading opportunities to each individual; these offers constitute the regime.

Certain kinds of trading institutions may be able to construct opportunity sets in such a way that, at least in an appropriately-defined equilibrium, the jointly chosen acquisition profile is feasible. Clearly, this is true of any trading institution that is able to set market-clearing prices at which individuals are allowed to trade freely, as in the familiar model of the Walrasian auctioneer or as in a Nash equilibrium of the arbitrage model presented by Sugden (2004). But there are other kinds of feasible regime, for example the regime in which no trade is allowed. Opportunity-based normative analysis, as pursued in the current paper, is concerned with the evaluation of alternative feasible regimes for a given economy.

## Opportunity criteria

It might seem that, in an opportunity-based analysis, the normative value of a regime should be defined as an aggregate of the values of the opportunities provided to individuals *separately* by their respective opportunity sets. It might then seem natural to state as a normative principle that ‘larger’ opportunity sets are more valuable than ‘smaller’ ones – or, more precisely, that the value of an opportunity set is always increased by the addition of a non-dominated option. Such a principle would be in the spirit of an important strand of literature on the measurement of opportunity (e.g. Kreps 1979; Jones and Sugden 1982; Pattanaik and Xu 1990; Arrow 1995). However, this approach is unsuitable for a normative assessment of alternative profiles of opportunity sets.

If the trading institution is to be interpreted merely as an intermediary in the transfer of goods between individuals, it must be assumed to be subject to the feasibility constraints encoded in *F*. If that institution offers a market-clearing regime, it is able to implement the acquisition vectors that individuals *in fact* choose from their opportunity sets; but it cannot guarantee to implement whatever acquisition vector each individual happens to choose from his opportunity set, irrespective of other individuals’ choices. On a literal interpretation of the Walrasian model of general equilibrium, it is not clear that points in an individual’s budget set other the point actually chosen are genuinely available to that individual. In a real market economy, of course, it is normally reasonable to assume that each individual is free to choose any point in his budget set, provided that other individuals’ choices are held constant; but that is because of contingent features of markets, such as the holding of inventories by firms, that are not represented in my model. Given that my normative analysis is intended to apply to regimes in general, it would be misleading to assume that every element of each individual’s opportunity set is a genuine option, irrespective of the specification of the relevant regime. My analysis is concerned with opportunities for individuals *collectively* to choose acquisition vectors that are feasible *in combination*.

The Opportunity Criterion, as proposed by McQuillin and Sugden (2012), is designed for this kind of analysis. It is a criterion against which, for a given exchange economy, any regime can be assessed:

*Opportunity Criterion*. A regime **O** satisfies the Opportunity Criterion if (i) **O** is market-clearing and (ii) for every feasible acquisition profile $\mathbf{q}\notin A(\mathbf{O})$, there is some individual $i\in I$ such that $\mathbf{q}_{i}$ is dominated in $O_{i}$.

To understand the normative intuition behind the Opportunity Criterion, consider a market-clearing regime **O**. If the Opportunity Criterion is not satisfied, there must be some feasible acquisition profile $\mathbf{q}^{\prime }$ that is non-dominated for every individual but which has *not* been made available. Clearly, no dominance-based argument can be deployed to show that, had that opportunity been made available in addition to those given by **O**, some individual would not have wanted to take it up. The implication is that individuals collectively lack the opportunity to make a combination of choices that conceivably they might all want to make and that is compatible with the resource constraints of the economy. The Opportunity Criterion requires that individuals are not deprived in this way.^9^

The Opportunity Criterion can be interpreted as an opportunity-based analogue of Pareto-optimality. To see the relationship between these criteria, consider a case in which individuals’ choices can be rationalised by preference orderings with the property that more money is always strictly preferred to less. In this case, to say that a regime induces a Pareto-optimal outcome is to say that, given individuals’ actual preferences, no feasible reallocation of commodities away from the jointly chosen acquisition profile $\mathbf{q}^*$ is Pareto-improving. To say that a regime satisfies the Opportunity Criterion is to say that every feasible reallocation of commodities away from $\mathbf{q}^*$ that individuals jointly could conceivably want to make *is in fact allowable to them*. The latter statement is stronger. For this reason, proving that a class of regimes satisfies the Opportunity Criterion can be a revealed-preference method of proving that that class induces Pareto optimality. Like the criterion of Pareto optimality, the Opportunity Criterion is silent on issues of distribution.

In proofs of the Pareto optimality of competitive equilibrium, it is normal to assume that individuals’ preferences satisfy some condition of non-satiation. In my analysis, the analogue of non-satiation is the role of dominance in the Opportunity Criterion. To see the necessity for some such analogue, consider the following two-person exchange economy. Individual 1 has well-behaved preferences over consumption bundles, with larger quantities of each commodity always preferred to less. Individual 2 has preferences with a bliss point at some consumption bundle that is strictly dominated by his endowment. To achieve Pareto optimality, there must be some unilateral transfer of goods from individual 2 to individual 1, but such a transfer cannot be made by trading at market-clearing prices. This anomalous case is normally excluded by assuming non-satiation. In my model, the Opportunity Criterion is defined in such a way that it is not contravened by the absence of an opportunity to make a dominated transaction. The definition of dominance is motivated by the implicit assumption that consumption of money is always valued positively.

To say that the Opportunity Criterion is satisfied is to say that *the set of all individuals* is not deprived of opportunities to make combinations of choices that are feasible and non-dominated. But that criterion has nothing to say about the presence or absence of opportunities for feasible combinations of choices by sets of individuals that do not contain everyone. The following definitions are preliminaries for formulating a stronger criterion.

Given the fixed exchange economy (which will be referred to as the *whole* economy), I define a *sub-economy* $<S, G, \mathbf{e}_{S}$, $\mathbf{f}_{S}(.)>$ for each nonempty set *S* of individuals (where S $\subseteq I$) by replacing arrays that refer to the whole economy (and sets of such arrays) by corresponding components (and sets of components) that are restricted to *S*. Such restricted arrays and sets are denoted by adding an *S* subscript to the entity that refers to the whole economy. An acquisition profile for *S*, $\mathbf{q}_{S}$, is *allowable in* $\mathbf{O}_{S}$ if each of its component acquisition vectors $\mathbf{q}_{i}$ is allowable in $O_{i}$; the set of profiles that are so allowable is $A_{S}(\mathbf{O}_{S})$. A profile $\mathbf{q}_{S}$ is *feasible* *for S* if, for each commodity $g,\sum _{i\in S} q_{i,g} = 0$. The set of feasible acquisition profiles for *S* is denoted $F_{S}$. Intuitively, this concept of feasibility treats the sub-economy for *S* as if there were no possibilities for transfers of commodities between those individuals who belong to *S* and those who do not. However, the concept of a sub-economy is to be understood merely a convenient formal device for representing particular features of the fixed economy.

The stronger criterion is:

*Strong Opportunity Criterion*. A regime **O** satisfies the Strong Opportunity Criterion if (i) **O **is market-clearing and (ii) for every nonempty set of individuals $S\subseteq I$, and for every acquisition profile $\mathbf{q}_{S}\in {\varvec{\mathscr {Q}}}_{S}$ for *S* such that $\mathbf{q}_{S }\notin A_{S}(\mathbf{O}_{S})$ and $\mathbf{q}_{S}\in F_{S}$, there is some individual $i \in S$ such that $\mathbf{q}_{i}$ is dominated in $O_{i}$.

In relation to the set $S=I$, this criterion imposes exactly the same restrictions as the Opportunity Criterion does; so any regime that satisfies the Strong Opportunity Criterion also satisfies the Opportunity Criterion. But the Strong Opportunity Criterion imposes restrictions analogous with those of the Opportunity Criterion for *every* set of individuals. It requires, for each such set *S*, that the members of *S* are not deprived of opportunities to make combinations of choices that are non-dominated and that are feasible within the resource constraints imposed by their combined endowments. Notice that, since a sub-economy is defined for each $\{i\}$, the Strong Opportunity Criterion requires that, for each *i*, the acquisition vector $\mathbf{q}_{i} = \mathbf{0}$ is allowable in $O_{i}$. That is, it requires that each individual has the opportunity to consume exactly the bundle of goods he was endowed with.

The Strong Opportunity Criterion can be interpreted as an opportunity-based analogue of the core. In a preference-based analysis, a jointly-chosen acquisition profile $\mathbf{q}^*$ is in the core of the relevant economy if and only if it is Pareto-optimal in every sub-economy. Thus, the Strong Opportunity Criterion is analogous with the core in the same sense that the Opportunity Criterion is analogous with Pareto optimality.

## Market opportunity theorems

I now characterise a particular type of regime for an exchange economy – a *single-price regime*. In such a regime, for each non-money commodity $g= 2, {\ldots }, m$, there is a *market price* $p_{g}$ expressed in money units; this price is finite, and may be positive, zero or negative. Each individual is free to keep his endowments if he chooses, but is also free to exchange claims on non-money commodities for claims on money (and vice versa) on terms that are at least as favourable as those implied by market prices, subject to the constraint that his holdings of claims on any commodity cannot be negative. Since all exchanges take place through the medium of money, there is no real meaning to the concept of a ‘market price of money’, but as a notational convention I define $p_{1} \equiv 1$.^10^ Formally, a single-price regime is defined by:

*Single-price regime*. A regime **O** is a single-price regime if there exists a finite, real-valued price vector $\mathbf{p} = (p_{1}, {\ldots }, p_{m})$ where $p_{1} = 1$, such that for each individual $i \in I$, every acquisition vector $\mathbf{q}_{i} \in {\varvec{\mathscr {Q}}}_{i}$ that satisfies $\Sigma _{g} p_{g} q_{i,g} = 0$ is either allowable or dominated in $O_{i}$.

A single-price regime is *strict* if, for each *i*, $O_{i}$ contains all acquisition vectors that satisfy $\Sigma _{g}p_{g} q_{i,g} = 0$, and no others.

A market-clearing single-price regime describes the opportunities available in a competitive equilibrium. Notice that the definition of a market-clearing single-price regime allows the possibility that individuals have opportunities to trade on more favourable terms than those specified by **p**. If individuals were assumed not to choose dominated acquisition vectors, market-clearing would be possible only if opportunities of this kind were not in fact taken up: all *actual* trades would take place at exactly the prices specified by **p**.^11^ Nevertheless, it is necessary to take account of the possibility of such favourable but non-chosen opportunities when considering what properties of regimes are implied by an opportunity-based criterion.

The idea of a single-price market-clearing regime is an equilibrium concept. Its usefulness depends on an implicit assumption that the trading institution can set prices that clear markets. But is there any reason to expect that a market-clearing equilibrium exists? In Appendix 2 I show that a market-clearing single-price regime exists for every exchange economy that satisfies specific assumptions about how, in general, choices respond to changes in prices.^12^ In classic proofs of the existence of competitive equilibrium, properties of demand functions derive from fundamental assumptions about the properties of rational preferences and about the relationship between preference and choice. In my modelling framework, in contrast, there is no concept of preference and there are no consistency restrictions on choice. The additional assumptions used in my existence theorem can be justified only as plausible empirical generalisations. In this sense, the theorem is not analogous with classic existence theorems. Nevertheless, it gives some support to the intuitive idea that competition tends to induce market-clearing, even when individuals do not act on consistent preferences. In the main text of this paper, however, I simply examine the properties of market-clearing single-price regimes.

My first main result (proved in Appendix 1) is the following:

*Strong Market Opportunity Theorem*. For every exchange economy, every market-clearing single-price regime satisfies the Strong Opportunity Criterion.

As a corollary of this, I obtain a marginal strengthening of a result proved by McQuillin and Sugden (2012):

*Market Opportunity Theorem*. For every exchange economy, every market-clearing single-price regime satisfies the Opportunity Criterion.

The Market Opportunity Theorem is the opportunity-based analogue of the First Fundamental Theorem of Welfare Economics, as applied to exchange economies. The Strong Market Opportunity Theorem is the analogue of the theorem that every competitive equilibrium of an exchange economy is in the core.

## The opportunity convergence theorem

Not all regimes satisfying the Strong Opportunity Criterion are single-price regimes:

### Proposition 1

There exists an exchange economy and a non-single-price regime for that economy such that the Strong Opportunity Criterion is satisfied.

The example that I use to establish this result is a two-person, two-commodity exchange economy in which both individuals have non-zero endowments of both commodities. Consider a regime **O** for such an economy in which there are two different (strictly positive) prices at which commodity 2 can be traded – a high price ${p_{2}}^{\mathrm{H}}$ and a low price ${p_{2}}^{\mathrm{L}}$. Individual 1 is allowed to buy commodity 2 at the low price or sell at the high price (but not both), while the opposite is true of individual 2. The market clears at the high price, with individual 2 buying from individual 1 (i.e., $q_{2,2} = -q_{1,2}> 0$). I will call this the *two-price Edgeworth box regime*. To aid intuition about later results I now show diagrammatically that this regime does not satisfy the definition of a single-price regime. I complete the proof of Proposition 1 in Appendix 1, where I show that this regime satisfies the Strong Opportunity Criterion.

**Fig. 1:**
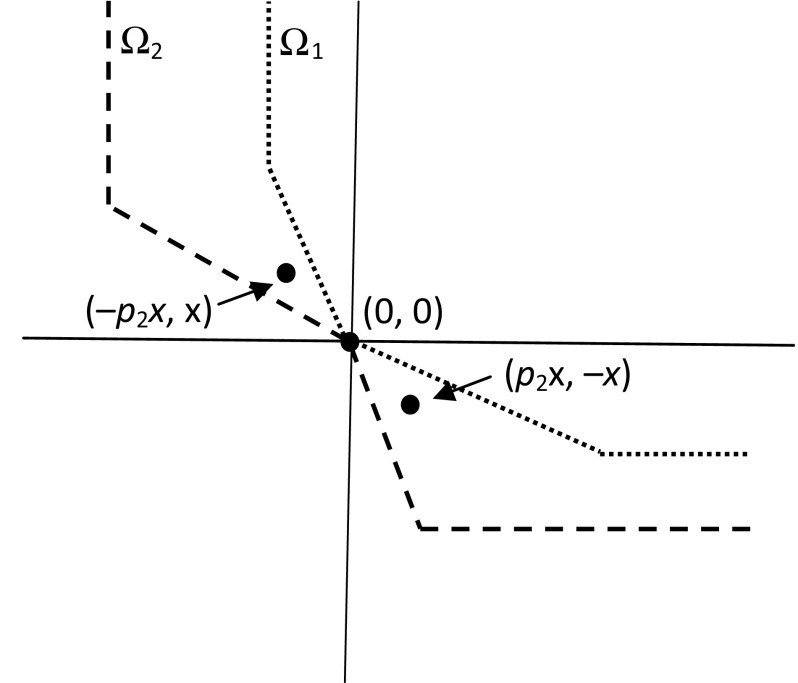
Non-allowable, non-dominated acquisition vectors in a two-price Edgeworth box regime

For each individual $i = 1, 2$ facing the two-price Edgeworth box regime **O**, let $\Omega _{i}$ be the set of acquisition vectors $\mathbf{q}_{i} \in \varvec{\mathscr {Q}}_{i}$ that are neither allowable nor dominated in $O_{i}$. Figure 1 plots $\Omega _{1}$ and $\Omega _{2}$ in a diagram in which the horizontal axis measures acquisition (by either individual) of commodity 1, and the vertical axis measures acquisition (by either individual) of commodity 2. $\Omega _{1}$ is the set of points above and to the right of the *dotted* frontier; $\Omega _{2}$ is the set of points above and to the right of the *dashed* frontier. The horizontal and vertical segments of these frontiers reflect the constraint that each individual’s consumption of each commodity must be non-negative (i.e., for each *i* and $g,q_{i,g} \ge -e_{i,g}$).

Now (to initiate a proof by contradiction) suppose that **O** is a single-price regime. By the definition of such a regime, there exists a finite price $p_{2}$ such that every acquisition vector $\mathbf{q}_{2}$ that satisfies $q_{2,1}+p_{2} q_{2,2}= 0$ is either allowable or dominated in $O_{2}$. This is equivalent to saying that the line through **0** with gradient $-1/p_{2}$ does not pass through $\Omega _{2}$. But it is immediately obvious from the diagram that, whatever the value of $p_{2}$, this line *does *pass through $\Omega _{2}$. So the supposition that **O** is a single-price regime is false. But, as I show in Appendix 1, **O** satisfies the Strong Opportunity Criterion.

However, there is a sense in which, for a sufficiently large economy, any regime that satisfies the Strong Opportunity Criterion is ‘almost’ a single-price regime. I formalise this idea in terms of replica economies, as first used by Edgeworth (1881/ 1967). This method of analysis works by taking some economy, replicating every component of it, and then creating a larger economy by combining two or more of these replicas. The beauty of this method is that, despite the artificiality of assuming exact replication, it allows one to investigate the effect of changing the scale of an economy while holding all other features constant. In a preference-based model, preferences are held constant as individuals are replicated. In my model, correspondingly, choice functions are held constant as individuals are replicated.

Formally, I fix an exchange economy $E=<I, G, \mathbf{e}, \mathbf{f}(.)>$ with individuals $i = 1, {\ldots }, n$. For each integer $r \ge 1$, the *r-fold economy* $E^{r}$ is defined as the exchange economy created by combining *r* replicas of *E*. Its joint choice function $\mathbf{f}^{r}(.)$ replicates **f**(.) in a corresponding way. Similarly, for any regime **O **for economy *E*, the *r-fold regime* $\mathbf{O}^{r}$ is defined as the regime that combines *r* replicas of **O**. Thus, $E^{r}$ is an economy with *rn* individuals. For each $k = 1, {\ldots },n$, each of the individuals $k,k +r,k + 2r, {\ldots }, k + (n- 1)r$ in economy $E^{r}$ has the same endowment vector $\mathbf{e}_{k}$, the same choice function $\mathbf{f}_{k}$, and the same opportunity set $O_{k}$ as does individual *k* in economy *E* with regime **O**. Thus $E^{1} \equiv E$ and $\mathbf{O}^{1} \equiv \mathbf{O}$.

As an illustration of the significance of replication for the Strong Opportunity Criterion, consider the two-fold replica ($E^{2}, \mathbf{O}^{2}$) of the economy in Fig. 1. Recall that ($E, \mathbf{O}$) satisfies the Strong Opportunity Criterion (i.e., in economy *E*, regime **O** satisfies that criterion). **O** is market-clearing in *E* and so, by virtue of the definition of replication, $\mathbf{O}^{2}$ is market-clearing in $E^{2}$. However, ($E^{2}, \mathbf{O}^{2}$) does not satisfy the Strong Opportunity Criterion. This is because, in the two-fold economy, individuals 2 and 4 (who are replicas of one another) are deprived of opportunities for trade *between themselves* that are feasible *for them*. To see this, consider any price $p_{2}\in ({p_{2}}^{\mathrm{L}}, {p_{2}}^{\mathrm{H}})$ and any *x* such that $e_{2,1}=e_{4,1} \ge p_{2} x$ and $e_{2,2}=e_{4,2} \ge x$. Consider the acquisition profile $\mathbf{q}_{\{2,4\}}$ where $\mathbf{q}_{2 }= (-p_{2}x, x)$ and $\mathbf{q}_{4 }= (p_{2}x,-x)$. Clearly, this acquisition profile is feasible for the set of individuals $\{2, 4\}$. But $\mathbf{q}_{2 }\in \Omega _{2}$ and $\mathbf{q}_{4} \in \Omega _{4}$, which violates the Strong Opportunity Criterion.

This example illustrates a general property of replica economies: the larger the scale of an economy, the more difficult it is to find a market-clearing regime that satisfies the Strong Opportunity Criterion but is not single-price. In fact, in the limit as the scale of an economy increases, the only regimes that satisfy the Strong Opportunity Criterion are those that are ‘almost the same as’ single-price regimes.

Consider any pair ($E, \mathbf{O}$) of an exchange economy and a regime for that economy. For any individual *i*, for any acquisition vector $\mathbf{q}_{i }\in \varvec{\mathscr {Q}}_{i}$ and any finite real number ${\upvarepsilon } > 0$, let ${\uppsi }(\mathbf{q}_{i}, {\upvarepsilon })$ be the set of acquisition vectors whose Euclidian distance from $\mathbf{q}_{i }$ is no greater than ${\upvarepsilon }$. I will say that $\mathbf{q}_{i }$ is ‘within ${\upvarepsilon }$ of being allowable’, or ${\upvarepsilon }$ *-allowable*, in $O_{i}$ if there is some acquisition vector $\mathbf{q}^{\prime }_{i }\in {\uppsi }(\mathbf{q}_{i,} {\upvarepsilon })$ that is allowable in $O_{i}$, that $\mathbf{q}_{i }$ is ${\upvarepsilon }$ *-dominated* in $O_{i}$ if there is some acquisition vector $\mathbf{q}^{\prime }_{i }\in {\uppsi }(\mathbf{q}_{i}, {\upvarepsilon })$ that is dominated in $O_{i}$, and that **O** is an ${\upvarepsilon }$ *-single-price regime* if there exists a finite, real-valued price vector $\mathbf{p} = (p_{1}, {\ldots }, p_{m})$ with $p_{1} = 1$ such that, for each individual *i*, every acquisition vector $\mathbf{q}_{i}$ that satisfies $\Sigma _{g} p_{g} q_{i,g} = 0$ is either ${\upvarepsilon }$-allowable in $O_{i}$ or ${\upvarepsilon }$-dominated in $O_{i}$. Thus, at sufficiently small values of ${\upvarepsilon }$, ${\upvarepsilon }$-single-price regimes are ‘almost the same as’ single-price regimes. Then:

*Opportunity Convergence Theorem*. Let (*E*, **O**) be any pair of an exchange economy and a regime for that economy. *If* for all $r \ge 1$, the *r*-fold replica ($E^{r}, \mathbf{O}^{r}$) satisfies the Strong Opportunity Criterion, *then* for every finite ${\upvarepsilon } >0$, **O** is a market-clearing ${\upvarepsilon }$-single-price regime.

This result is an opportunity-based analogue of the Core Convergence Theorem (Edgeworth, 1881/1967; Debreu and Scarf 1963; Aumann 1964). However, the two theorems are conceptually distinct. The Core Convergence Theorem is about the relationship between the *allocations *(i.e., profiles of consumption bundles) induced by competitive equilibrium and a preference-based normative criterion. The Opportunity Convergence Theorem is about the relationship between the *opportunity profiles* induced by competitive equilibrium and a normative criterion that does not refer to preferences.

## Conclusion

Taken together, the Strong Market Opportunity Theorem and the Opportunity Convergence Theorem characterise a market-clearing single-price regime for a sufficiently large exchange economy. A market-clearing single-price regime can be interpreted as representing the opportunities that are made available to individuals in a competitive equilibrium. In the limit as the size of the economy tends to infinity, such a regime, and only such a regime, satisfies the Strong Opportunity Criterion.

I submit that this characterisation says something important about the opportunity-enhancing properties of competitive equilibrium that does not depend on any assumptions about the coherence of individuals’ preferences. Intuitively, the Strong Opportunity Criterion requires that, for any set of individuals in an economy, every transaction that those individuals might reasonably want to make and that is feasible, given their endowments, is available to them in their respective opportunity sets. Let us say that a person is *willing to pay for* something if he is willing to give up enough of his endowments to make others willing to play their parts in supplying it to him. A regime that satisfies the Strong Opportunity Criterion, one can then say, allows every individual to get whatever he wants and is willing to pay for. That every competitive equilibrium has this property, and that every regime that has this property is fundamentally similar to competitive equilibrium, are normatively significant statements.

## Appendix 1: Proofs of results in main text

### Proof of strong market opportunity theorem

It is convenient to use the following Lemma:

#### Lemma 1

Consider any exchange economy and any market-clearing regime **O** for that economy. Suppose that, for each individual *i*, there is some function $v_{i}: \varvec{\mathscr {Q}}_{i} \rightarrow \mathbb {R}$ satisfying the following three conditions:

(i) for all $\mathbf{q} \in \varvec{\mathscr {Q}}$, for every nonempty $S \subseteq I: \mathbf{q}_{S} \in F_{S} \Rightarrow \sum _{i\in S} v_{i}(\mathbf{q}_{i}) = 0$;
(ii) for all $i \in I$, for every $\mathbf{q}_{i}\in \varvec{\mathscr {Q}}_{i}$: *if* $v_{i}(\mathbf{q}_{i}) \le 0$, *then* *either* $\mathbf{q}_{i}$ is allowable in $O_{i}$ *or* $\mathbf{q}_{i}$ is dominated in $O_{i}$; and
(iii) for every $i \in I$, for every $\mathbf{q}_{i} \in \varvec{\mathscr {Q}}_{i}$: *if* $v_{i}(\mathbf{q}_{i}) < 0$, *then* $\mathbf{q}_{i}$ is dominated in $O_{i}$.

Then **O** satisfies the Strong Opportunity Criterion.

#### Proof of Lemma 1

Consider any exchange economy, any market-clearing regime **O** for that economy, and any list $v_{1}, {\ldots }, v_{n}$ of functions satisfying conditions (i), (ii) and (iii). Consider any nonempty set $S \subseteq I$ of individuals, and any acquisition profile $\mathbf{q}_{S} \in \varvec{\mathscr {Q}}_{S}$ for *S* such that $\mathbf{q}_{S}\in F_{S}$ and $\mathbf{q}_{S} \notin A_{S}(\mathbf{O}_s)$. By (i), $\sum _{i\in S} v_{i}(\mathbf{q}_{i}) = 0$. Thus, *either* $v_{i}(\mathbf{q}_{i}) = 0$ for all $i \in S$ (Case 1) *or* $v_{j}(\mathbf{q}_{j}) <0$ for some $j \in S$ (Case 2). In Case 1, by (ii), for each $i \in S$, $\mathbf{q}_{i}$ is either allowable in $O_{i}$ or dominated in $O_{i}$. Since $\mathbf{q}_{S} \notin A_{S}(\mathbf{O}_s)$, there must be some $i \in S$ such that $\mathbf{q}_{i }$ is dominated in $O_{i}$. In Case 2, by (iii), there is some $i \in S$ such that $\mathbf{q}_{i }$ is dominated in $O_{i}$. Since these results hold for every nonempty $S \subseteq I$, the Strong Opportunity Criterion is satisfied. $\square$

#### Proof of theorem

Suppose that, for some exchange economy, **O** is a market-clearing single-price regime. Since **O** is a single-price regime, there exists a finite, real-valued price vector $\mathbf{p} = (p_{1}, {\ldots }, p_{m})$ with $p_{1} = 1$ such that, for each individual *i*, every acquisition vector $\mathbf{q}_{i}$ that satisfies $\Sigma _{g} p_{g} q_{i,g} = 0$ is either allowable or dominated in $O_{i}$. For each $i \in I$, define $v_{i}(\mathbf{q}_{i})=\Sigma _{g} p_{g} q_{i,g}$. It is immediate that this definition satisfies conditions (i), (ii) and (iii) of Lemma 1. $\square$

### Proof of Proposition 1

In Sect. 6, I exhibited an exchange economy and a ‘two-price Edgeworth box regime’ for that economy. I proved that this is not a single-price regime. To prove Proposition 1, it is sufficient to show that this regime satisfies the Strong Opportunity Criterion. The regime can be characterised by using functions $v_{i}: \varvec{\mathscr {Q}}_{i} \quad \rightarrow \mathbb {R}$ for $i = 1, 2$, defined as follows (with ${p_{2}}^{\mathrm{L}} < {p_{2}}^{\mathrm{H}}$):

1. $v_{i}(\mathbf{q}_{i})=q_{i,1}+{p_{2}}^{\mathrm{L} }q_{i,2 }$ *if either* ($i = 1$ *and* $q_{i,2} \ge 0$) *or* ($i = 2$ *and* $q_{i,2} <0$) and
2. $v_{i}(\mathbf{q}_{i})=q_{i,1}+{p_{2}}^{\mathrm{H} } q_{i,2 }$ *if either* ($i = 1$ *and* $q_{i,2}<0$) *or* ($i = 2$ *and* $q_{i,2} \ge 0$).

(Intuitively, $v_{i}(\mathbf{q}_{i})$ is the total net value of *i*’s acquisitions, calculated at the prices set for *i* by the regime, for any acquisition vector $\mathbf{q}_{i}$.) Then, for each *i*, the regime is defined by $O_{i} = \{\mathbf{q}_{i}: \mathbf{q}_{i} \in \varvec{\mathscr {Q}}_{i}$ *and* $v_{i}(\mathbf{q}_{i}) = 0\}$. It is easy to show that these $v_{i}$ functions satisfy conditions (i), (ii) and (iii) of Lemma 1, and hence that the Strong Opportunity Criterion is satisfied. $\square$

### Proof of opportunity convergence theorem

Let ($E, \mathbf{O}$) be any pair of an exchange economy and a regime for that economy and let $I = \{1, {\ldots }, n\}$ be the set of individuals in that economy. For each $r \ge 1$, let ($E^{r}, \mathbf{O}^{r})$ be the corresponding *r*-fold economy and regime, with the set of individuals $I^{r} = \{1, {\ldots }, { rn}\}$. Suppose that the Strong Opportunity Criterion holds for every such *r*-fold economy. By the definition of that criterion, every $\mathbf{O}^{r}$ is market-clearing.

Fix any finite ${\upvarepsilon } > 0$. For each individual *i*, let $\Omega _{i}$ be the set of acquisition vectors for *i* that are neither allowable nor dominated in $O_{i}$. Each of these sets can be plotted in a common space $\mathbb {R}^{m }$ where *m* is the number of goods in the economy. (Each dimension $g = 1, {\ldots }, m$ of this space measures increments of good *g*, but without referring to any particular individual. Compare Figure 1.) Let $\Omega$ be the convex hull of the sets $\Omega _{i}$ for $i = 1, {\ldots }, n$. Let $\Omega ^*_{i}$ (${\upvarepsilon }$) be the set of acquisition vectors for *i* that are neither ${\upvarepsilon }$-allowable nor ${\upvarepsilon }$-dominated in O$_{i}$. Let $\Omega ^*({\upvarepsilon })$ be the convex hull of the sets $\Omega ^*_{i}$ (${\upvarepsilon }$) for $i = 1, {\ldots }, n$. By the definitions of ${\upvarepsilon }$-allowability and ${\upvarepsilon }$-dominance, for each *i*, every element of $\Omega ^*_{i}$ (${\upvarepsilon }$) is strictly in the interior of $\Omega _{i}^{.}$.

Consider any ${\upomega } \in \Omega ^*({\upvarepsilon })$. By the definition of a convex hull, ${\upomega }$ can be constructed as a convex combination of some finite set of vectors $\{{\upomega }_{1},{\ldots }, {\upomega }_{N}\}$ where each ${\upomega }_{j}$ is an element of $\Omega ^* _{i}({\upvarepsilon })$ for some individual $i \in I$ and each ${\upomega }_{j}$ has a real-valued weight ${\upalpha }_{j }$ in this combination, where $0<{\upalpha }_{j} \le 1$ and $\Sigma _{j }{\upalpha }_{j}= 1$. I will say that *i* is the *actor* for ${\upomega }_{j}$, and write this as $i =a(j)$. The ${\upalpha }_{j}$ weights need not be rational numbers. However, because the rational numbers form a dense subset of the reals, we can find vectors ${\upomega }^{\prime }_{1}, {\ldots }, {\upomega }^{\prime }_{N}$, such that each ${\upomega }^{\prime }_{j}$ is sufficiently close to the corresponding ${\upomega }_{j }$ that it is an element of $\Omega _{a(j)}$, and such that ${\upomega }$ is a convex combination of ${\upomega }^{\prime }_{1}, {\ldots }, {\upomega }^{\prime }_{N}$ in which each of the weights ${\upalpha }^{\prime }_{1}, {\ldots }, {\upalpha }^{\prime }_{N}$ is a strictly positive *rational* number.

Now consider, for some $r \ge 1$, the *r*-fold economy and regime ($E^{r}, \mathbf{O}^{r})$ that corresponds with ($E, \mathbf{O}$). The set of individuals in this economy is $I^{r} = \{1, {\ldots }, { rn}\}$. By virtue of the results established in the previous paragraph, if *r* is sufficiently large, we can construct a non-empty set $S \subseteq I^{r}$ of individuals, and an acquisition profile $\mathbf{q}_{S}$ for *S*, such that *S* can be partitioned into non-empty subsets $S_{1}, {\ldots }, S_{N}$ and the following properties hold for each $j = 1, {\ldots }, N$: (i) the ratio between the number of individuals in $S_{j}$ and the number of individuals in *S* is ${\upalpha }^{\prime }_{j}$; (ii) each individual in $S_{j}$ is a replica of the individual *a*(*j*); and (iii) the acquisition vector $\mathbf{q}_{i}$ for each individual *i* in $S_{j}$ is ${\upomega }^{\prime }_{j}$. (Note that, because ${\upomega }^{\prime }_{j}$ is an element of $\Omega _{a(j)}$, ${\upomega }^{\prime }_{j} \in \varvec{\mathscr {Q}}_{a(j)}$. Thus it is legitimate to treat the vector ${\upomega }^{\prime }_{j}$ as an acquisition vector for any replica of *a*(*j*).) Given these properties, $\mathbf{q}_{S}$ is feasible for *S* if and only if ${\upomega } = \mathbf{0}$.

Now suppose that $\mathbf{0} \in \Omega ^*({\upvarepsilon })$. Recall that, for each $i = 1, {\ldots }, n, \Omega ^* _{i}({\upvarepsilon }) \subset \Omega _{i}^{.}$. Applying the conclusions of the previous paragraph to the case ${\upomega } = \mathbf{0}$, if *r* is sufficiently large, there exists a non-empty set $S \subseteq I^{r}$ of individuals, and a feasible acquisition profile $\mathbf{q}_{S}$ for *S*, such that, for each $i \in S,\mathbf{q}_{i} \in \Omega _{i}$ (i.e., $\mathbf{q}_{i}$ is neither allowable nor dominated in $O^{r}_{i}$). This implies that ($E^{r}, \mathbf{O}^{r})$ does not satisfy the Strong Opportunity Criterion, contradicting an initial supposition of the proof. Thus, $\mathbf{0} \notin \Omega ^*({\upvarepsilon })$.

Since $\Omega ^* ({\upvarepsilon })$ is a convex set by construction, $\mathbf{0} \notin \Omega ^* ({\upvarepsilon })$ implies that there is some hyperplane through **0** that does not intersect $\Omega ^* ({\upvarepsilon })$, and hence does not intersect any $\Omega ^* _{i}({\upvarepsilon })$. Thus, by the definition of $\Omega _{i}^*({\upvarepsilon })$, for each individual $i = 1, {\ldots }, n$, every acquisition vector $\mathbf{q}_{i} \in \varvec{\mathscr {Q}}_{i}$ on this hyperplane is either ${\upvarepsilon }$-allowable or ${\upvarepsilon }$-dominated in $O_{i}$. Equivalently, there exists a finite, real-valued price vector $\mathbf{p} = (p_{1}, {\ldots }, p_{m})$ with $p_{1} = 1$ such that, for each individual *i*, every acquisition vector $\mathbf{q}_{i}$ that satisfies $\Sigma _{g} \quad p_{g} \quad q_{i,g} = 0$ is either ${\upvarepsilon }$-allowable or ${\upvarepsilon }$-dominated in $O_{i}$, i.e.,, **O** is an ${\upvarepsilon }$-single-price regime. $\square$

## Appendix 2: existence of a market-clearing single-price regime

Consider any exchange economy. For every finite price vector $\mathbf{p} = (p_{1}, {\ldots }, p_{m})$ with $p_{1} = 1$, there is a corresponding strict single-price regime **O**, defined (for each individual *i*) by $O_{i}= \{\mathbf{q}_{i}: \mathbf{q}_{i}\in \varvec{\mathscr {Q}}_{i}\ { and}\ \Sigma _{g} p_{g} q_{i,g} = 0\}$. Thus, considering only strict single-price regimes, the chosen acquisition vector $\mathbf{q}_{i}$ of each individual *i* can be expressed as a function of **p**. And so, for each commodity $g = 1, {\ldots }, m$, we can write *net excess demand* for *g* (i.e., the sum of the chosen values of $q_{i,g}$ for all individuals *i*) as a function $x_{g}(\mathbf{p})$. For any given price vector **p**, the corresponding strict single-price regime is market-clearing if and only if $x_{g}(\mathbf{p}) = 0$ for every commodity *g*. Notice that if $x_{g}(\mathbf{p}) = 0$ holds for all *non-money* commodities $g = 2, {\ldots }, m$, it necessarily holds for money too. More generally, *the value of* net excess demand, expressed in money units by using the price vector **p** and summed over all individuals and all commodities, is identically equal to zero, irrespective of whether markets clear. That is, $\Sigma _{i} \Sigma _{g} p_{g }x_{g}(\mathbf{p}) = 0$. This identity (a version of *Walras’s Law*) is an implication of the assumption that each individual’s chosen acquisition vector is in his opportunity set; it does not depend on any assumptions about preferences.

Now consider the following two additional assumptions:
*Continuity*. For each non-money commodity $g = 2, {\ldots }, m, x_{g}(\mathbf{p})$ is a continuous function.
*Intrinsic Value of Money*. For each non-money commodity $g = 2, {\ldots }, m$, there is an *upper limit* price ${p_{g}}^{\mathrm{U}} > 0$ and a *lower limit* price ${p_{g}}^{\mathrm{L}} <0$, such that, for all price vectors **p**, $p_{g} \ge {p_{g}}^{\mathrm{U}}$ implies $x_{1}(\mathbf{p}) >0$, and $p_{g} \le {p_{g}}^{\mathrm{L}}$ implies $x_{1}(\mathbf{p}) >0$. Since I am not assuming that individuals act on coherent preferences, I cannot follow the neoclassical strategy of deriving Continuity as a property of the demand functions of rational individuals whose preferences are ‘well-behaved’. However, I suggest that Continuity is a plausible assumption about *aggregate* behaviour in a large economy. Intrinsic Value of Money expresses the idea that money is always perceived as a desirable consumption good, and that this desire is never satiated. Intuitively, if the price of some non-money commodity *g* is sufficiently high, individuals who have positive endowments of *g* will want to take advantage of the opportunity to acquire large amounts of money by giving up small amounts of *g*, and so money will be in excess demand. Similarly, if the price of some non-money commodity *g* is sufficiently negative, individuals will want to take advantage of the opportunity to acquire large amounts of money by taking on small amounts of *g*, and so again money will be in excess demand. Because I have not assumed that every individual has a positive endowment of every commodity, I cannot justify Intrinsic Value of Money as a natural property of each individual’s demands, considered separately; it is merely a plausible assumption about aggregate behaviour.

The following theorem can be proved:
*Existence Theorem*. For any exchange economy, if Continuity and Intrinsic Value of Money are satisfied, there exists a market-clearing strict single-price regime.

### Proof

Consider any exchange economy. Assume that Continuity and Intrinsic Value of Money are satisfied. Let *P* be the set of price vectors **p** that satisfy the condition that, for each non-money commodity $g = 2, {\ldots }, m, {p_{g}}^{\mathrm{U}} \ge p_{g} \ge {p_{g}}^{\mathrm{L}}$. For any such price vector **p**, for each commodity $g = 1, {\ldots }, m$, let $x_{g}(\mathbf{p})$ be the net excess demand for commodity *g* that would occur if **p** was the price vector in a strict single-price regime. I now define a *tâtonnement function* ${\varvec{\zeta }}: P \rightarrow P$. For the purposes of the proof, this is merely a mathematical construction.

As a first step, I define ${\upphi }(z)={\upalpha }(1- e^{-z})/(1 + e^{-z})$ for all real numbers *z*, where *e* is Euler’s number and ${\upalpha }$ is some constant satisfying $1 \ge {\upalpha } > 0$. Notice that ${\upphi }$(.) is a continuous and monotonically increasing function with ${\upphi }(0) = 0; {\upphi }(z) \rightarrow -{\upalpha }$ as $z \rightarrow -\infty$, and ${\upphi }(z) \rightarrow {\upalpha }$ as $z \rightarrow \infty$. Writing ${\varvec{\zeta }}(\mathbf{p})$ as [$\zeta _{1}(\mathbf{p}), {\ldots }, \zeta _{m}(\mathbf{p})$], I define $\zeta _{g}(\mathbf{p})$ for $g = 2, {\ldots }, m$ by:

1. $$\zeta _{g}(\mathbf{p}) = (1 -{\upphi }[x_{g}(\mathbf{p})])p_{g}+{\upphi }[x_{g}(\mathbf{p})]{p_{g}}^{\mathrm{U}}\ { if}\ x_{g}(\mathbf{p}) \ge 0$$; *and*
2. $$\zeta _{g}(\mathbf{p}) = (1 -{\upphi }[x_{g}(\mathbf{p})])p_{g}+{\upphi }[x_{g}(\mathbf{p})]{p_{g}}^{\mathrm{L}}\ { if}\ x_{g}(\mathbf{p}) \le 0$$.

Because of Walras’s Law, these equations also define $\zeta _{1}(\mathbf{p})$. Because ${\upphi }$(.) is a continuous function, and because (by Continuity) each $x_{g}$(.) is a continuous function, ${\varvec{\zeta }}(.)$ is a continuous function from *P* to *P*. By construction, *P* is a closed, bounded, convex set. Thus, by Brouwer’s Theorem, there is a fixed point $\mathbf{p}^* \in P$ such that ${\varvec{\zeta }}(\mathbf{p}^*) = \mathbf{p}^*$.

Consider any such $\mathbf{p}^*$. First, suppose there is some non-money commodity *g* such that *either* $p^*_{g}={p_{g}}^{\mathrm{U}}$ *or* $p^*_{g}={p_{g}}^{\mathrm{L}}$. So, by Intrinsic Value of Money, $x_{1}(\mathbf{p}^*) >0$. ByWalras’s Law, there must be some non-money commodity *h* (which may or may not be *g*) for which the value of net excess demand is strictly negative, i.e., $p^*_{h}x_{h}(\mathbf{p}^*) < 0$. By the definition of $\mathbf{p}^*, \zeta _{h}(\mathbf{p}^*) = p^*_{h}$. Thus, *either* $p^*_{h }>0$ and $x_{h}(\mathbf{p}^*) <0$ (Case 1), *or* $p^*_{h }<0$ and $x_{h}(\mathbf{p}^*) >0$ (Case 2). Suppose Case 1 holds. By (A2), [$\zeta _{h}(\mathbf{p}^*) = p^*_{h }$ and $x_{h}(\mathbf{p}^*) <0$] implies $p^*_{h}={p_{h}}^{\mathrm{L}} <0$, a contradiction. Suppose Case 2 holds. By (A1), [$\zeta _{h}(\mathbf{p}^*) = p^*_{h }$ and $x_{h}(\mathbf{p}^*)>0$] implies $p^*_{h}={p_{h}}^{\mathrm{U}} >0$, a contradiction. So the original supposition is false. That is, for every non-money commodity $g,{p_{g}}^{\mathrm{U}}> p^*_{g} >{p_{g}}^{\mathrm{L}}$.

It then follows from (A1) and (A2) that, for each non-money commodity $g,\zeta _{g}(\mathbf{p}^*) = p^*_{g}$ implies $x_{g}(\mathbf{p}^*) = 0$. Thus, there exists a market-clearing strict single-price regime, namely the strict single-price regime in which the price vector is $\mathbf{p}^*$. $\square$

## Footnotes

1 The two papers use slightly different specifications of an exchange economy, and slightly different formulations of the Opportunity Criterion. The present paper follows McQuillin and Sugden (2012). For details about the differences between the two formulations, and reasons for preferring the later one, see the 2012 paper.

2 Throughout the paper, I use bold letters to refer to arrays of objects and to array-valued functions. Arrays that include one object for each individual are called ‘profiles’. I reserve the term ‘vector’ for arrays that include one price or one quantity for each commodity.

3 Even in a model in which all commodities are goods, one might want to define feasibility constraints as equalities, as does Aumann (1964). If feasibility were instead defined in terms of weak inequalities (thus assuming all commodities to be freely disposable goods), my results concerning the Opportunity Criterion and Strong Opportunity Criterion would still hold. (Whether or not these criteria are satisfied is unaffected by the allowability or non-allowability of dominated acquisition vectors, and so is unaffected by the presence or absence of free disposal options.)

4 In the ‘small world’ of a partial-equilibrium model, money might be interpreted as potential expenditure on all goods not explicitly represented in that model. On this interpretation, the idea that consumption of money is always valued positively is very close to the idea that increases in opportunity are always desirable.

5 ‘Contextual features’ are analogous with the concepts of ‘frames’ and ‘ancillary conditions’ used respectively by Salant and Rubinstein (2008) and Bernheim and Rangel (2009).

6 It is not strictly necessary for my analysis that behaviour is perfectly predictable at the individual level. All that is required is that aggregate behaviour is sufficiently predictable for the trading institution to set market-clearing prices.

7 Because of the simple structure of choice problems in an exchange economy, there are relatively few ways in which revealed preferences can contravene standard coherence assumptions. The storage economies analysed by McQuillin and Sugden (2012) allow ‘irrationality’ to take a much wider range of forms.

8 It would be possible to close the model by assuming an auctioneer or social planner who is a government employee, any positive or negative surplus from whose trading operations accrues to individuals as lump-sum benefits or taxes. Analogously, one might assume a set of traders employed by firms whose shares are entirely owned by individuals. However, the model is simpler and more transparent if the trading agency is entirely separate from the individuals.

9 An equivalent statement of part (ii) of the Opportunity Criterion, used by McQuillin and Sugden (2012), is that it is not possible to relax the constraints that **O** imposes on individuals (thus expanding the set of allowable outcomes) in such a way that some feasible outcome, not allowable in **O**, becomes both allowable and non-dominated.

10 In many general equilibrium models, every good is treated as having its own price, expressed in some abstract unit of account; the principle that only relative prices matter is then derived from the theorem that, given well-behaved preferences, excess demand functions are homogenous of degree zero in prices. Since my model has no concept of preference, this modelling strategy is not open to me. Simply to assume the absence of money illusion would be contrary to my aim of building a model that can accommodate non-rational regularities of individual behaviour. My model has only *m*–1 meaningful prices; $p_{1} \equiv 1$ is a notational convention, not a normalisation.

11 Given the assumption that dominated acquisition vectors are not chosen, the total value of all chosen acquisitions in a single-price regime, valued in terms of **p**, is non-negative; it is strictly positive if any individual trades on terms more favourable than are specified by **p**. Market-clearing requires that this total value is zero.

12 One role of these assumptions is to require that, as prices of non-money goods tend to infinity (or as prices of bads tend to minus infinity), there is excess demand for money. Intuitively, this restriction rules out configurations of demand which, in standard general equilibrium models, would imply a zero price of money in equilibrium.
